# A quasi-experimental study to improve health service quality: implementing communication and self-efficacy skills training to primary healthcare workers in two counties in Iran

**DOI:** 10.1186/s12909-021-02796-4

**Published:** 2021-07-06

**Authors:** Hossein Shahnazi, Marzieh Araban, Mahmood Karimy, Mansooreh Basiri, Ali Ghazvini, LAR Stein

**Affiliations:** 1 Department of Health Education and Promotion, Social Determinants of Health Research center, School of Public Health, Isfahan University of Medical Sciences, Isfahan, Iran; 2 Department of Health Education and Promotion, Social Determinants of Health Research Center, Public Health School, Ahvaz Jundishapur University of Medical Sciences, Ahvaz, Iran; 3 Department of Public Health, Social Determinants of Health Research Center, Saveh University of Medical Sciences, Saveh, Iran; 4 Social Determinants of Health Research Center, Saveh University of Medical Sciences, Saveh, Iran; 5 Chemical Injuries Research Center, System Biology and Poisoning Institute, Baqiyatallah University of Medical Sciences, Tehran, Iran; 6 Psychology Department, University of RI, Kingston, RI USA; 7 Behavioral & Social Sciences Department, Brown University School of Public Health, Providence, RI USA; 8 Rhode Island Training School, Department of Children, Youth & Families, Cranston, RI USA; 9 Center for Prisoner Health & Human Rights, Brown University Medical School, Providence, RI USA

**Keywords:** Communication skills, Self-efficacy, Primary healthcare, Client

## Abstract

**Background:**

Service satisfaction ratings from clients are a good indicator of service quality. The present study aimed to investigate the impact of communication skills and self-efficacy training for healthcare workers on clients’ satisfaction.

**Methods:**

A quasi-experimental study was conducted in health centers of Saveh University of Medical Science in Iran. Primary Healthcare (PHC; *N* = 105) workers and service recipients (*N* = 364) were randomly assigned to intervention and control groups. The intervention group received four 90-min training sessions consisting of lecture, film screening, role-playing, and discussion group. Before and 3 months after the intervention, a multi-part questionnaire (including demographics, self-efficacy and communication skills in PHC workers; and satisfaction questionnaire in service recipients) was completed by participants in both intervention and control groups.

**Results:**

PHC worker mean scores of self-efficacy and communication skills after the educational program were increased in the intervention group compared to the control group (*p* < 0.05). Also, mean satisfaction scores for service recipients of the intervention group (PHC workers) generally significantly increased compared to the control group (*p* < 0.001).

**Conclusions:**

The educational program improved the self-efficacy, and communication skills in health workers and improved client satisfaction overall. Our results support the application of self-efficacy and communication skills training for other medical groups who wish to improve clients satisfaction as an important health services outcome.

## Introduction

Service satisfaction is affected by service quality, quality of service delivery, and levels of service recipients’ expectation of service quality [1, 2]. Service satisfaction is a good indicator of service quality [2]. Measurement of service recipient satisfaction is a common method for evaluating the treatment quality in healthcare organizations [3]. Generally, the concept of satisfaction in providing health services refers to the feeling or attitude of service clients. There is a direct relationship between patient satisfaction and remaining in treatment [4].

Appropriate interpersonal communication between healthcare providers and recipients is an important determinant of clients satisfaction and compliance with healthcare guidelines [5]. Proper and effective communication between health personnel and patients has a positive impact on health and medical care and enhances patient satisfaction. Focusing only on technical aspects of health care may lead professionals to use ineffective communication methods (e.g., lack of eye contact, not listening fully to client or patient concerns), and thus key and major problems of patients are not clearly identified [6, 7]. Communication skills (CS) are crucial to professionals that come in direct contact with clients. Such skills convey respect, attention and empathy; and frequently include asking open questions, listening actively, and using intelligible words for patients in order to increase the effectiveness of medical interview and treatment process as well as patients’ satisfaction [8–10].

Today, health managers and planners around the world, particularly in developing countries are facing important challenges in responding the health care needs of the general population [11, 12]. In Iran, Primary Healthcare (PHC) workers are responsible for providing appropriate health education and services for the public [13, 14]. PHC workers prevent patients from being referred to clinics and hospitals by providing primary health care [15]. Therefore, the PHC workers’ ability to communicate effectively with individuals is an essential requirement for satisfaction and engagement of service recipients to promote health [14, 15]. Improving self-efficacy (SE) for communicating may assist in improving CS [6]. SE is the main element of the social-cognitive theory that refers to an individual’s belief or judgment about their ability to perform tasks and responsibilities [16]. Therefore, SE is an important factor for successful performance, and the skills that lead to successful performance [6, 17].

In Iran, primary healthcare coverage is offered to over 95 % of rural areas, but quality of care is the main concern of health policymakers. Since satisfaction is an important index of quality and performance of health care [13, 15] and given the lack of information on how CS and SE of health workers affect patient satisfaction, the present study aimed to evaluate the impact of an educational intervention, based on SE and CS, for PHC workers. Of particular interest was impact on the satisfaction of public health service recipients.

## Methods

### Design, procedure and the study sample

**Fig. 1:**
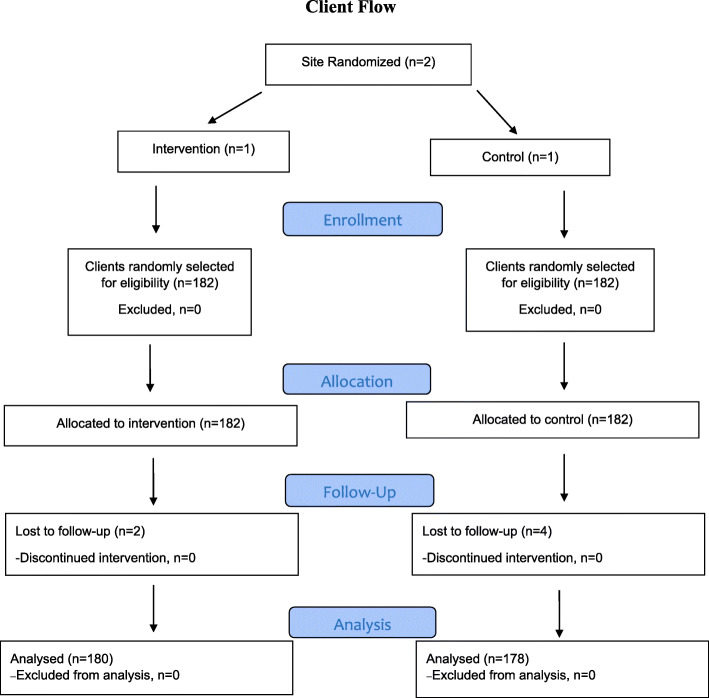
Consort Diagram

**Fig. 2:**
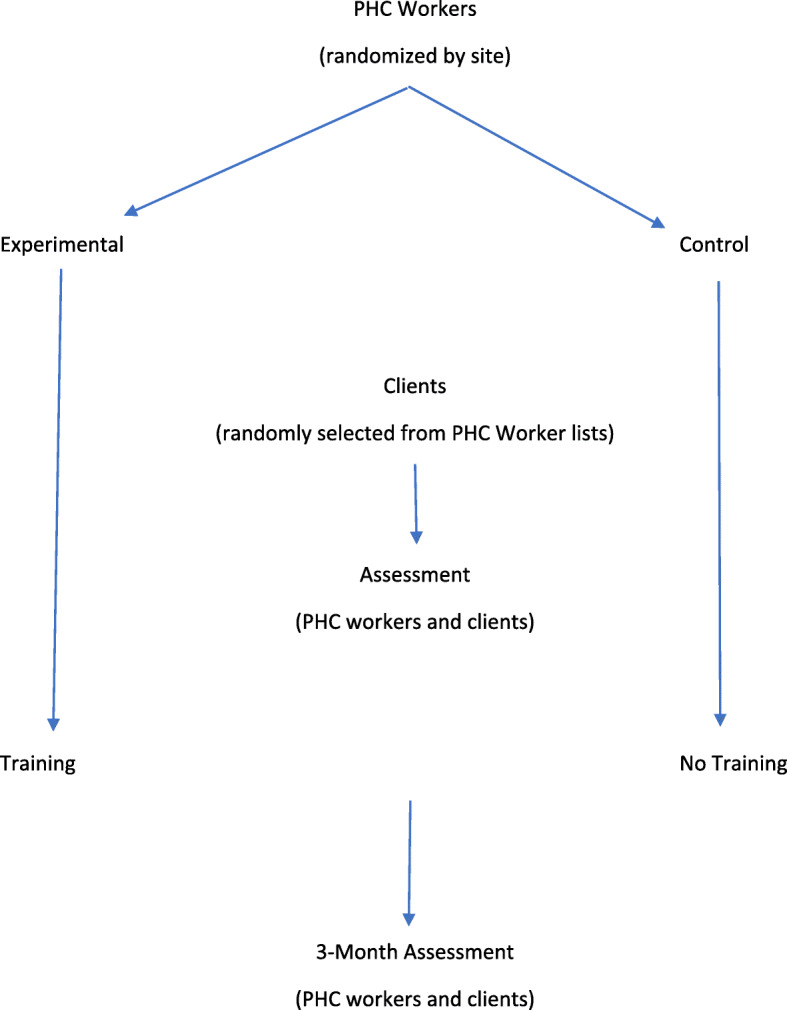
Study Design. PHC = Primary Healthcare

The present study, conducted in 2019, was a quasi-experimental intervention study conducted on primary healthcare workers (*N* = 105) in health centers of Saveh and Zarandieh counties, and patients (*N* = 364) living in rural areas of Saveh and Zarandieh. Setting power to 80 %, with a medium effect size and alpha = 0.01, the sample size needed for PHC workers was *N* = 44 per group (*N* = 88, in total), based on similar previous studies [17]. In anticipation of drop-out, *N* = 105 PHC workers were approached to participate in the study. One left just before beginning the study, leaving *N* = 104 PHC workers (*N* = 60 and *N* = 44 in intervention and control groups, respectively). Sample size needed for service recipients was calculated at *N* = 303 based on previous research [18], setting power to 80 %, with medium effect size and alpha = 0.01. Of the *N* = 364 service recipients screened for eligibility (see below) none were excluded, leaving *N* = 182 in both intervention and control conditions. Subsequently, *N* = 2 and *N* = 4 were lost to follow-up from intervention and control groups, respectively, leaving *N* = 358 for analyses (*N* = 180 and *N* = 178 in intervention and control groups, respectively). See Consort Diagram (Fig. 1). Figure 2 depicts study design.

Because PHC workers in Zarandieh and Saveh had similar scientific and cultural characteristics, PHC workers in Zarandieh were placed in the control group, and PHC workers in Saveh were placed in the intervention group. This was done by randomizing which site would be placed in control (using flip of a coin). Thereafter, personnel numbers were utilized to randomly sample PHC workers in each site. Service recipients were randomly selected (using random numbers table) from the list of clients seen by PHC workers in the last 3 months, and then were contacted and informed of the research purpose. Appointments took place at their homes where they completed the satisfaction questionnaire.

Inclusion criteria for PHC workers were anticipated continued employment for the next 6 months, at least one year of work experience (both determined through interview) and willingness to participate in the study. PHC workers were excluded if they were absent from two consecutive training sessions (see below). For service recipients, inclusion criteria were residence in Zarandieh or Saveh, receipt of PHC services in the last 3 months, being 15 years or older and willingness to participate in the study.

### Measures

A multi-part assessment included demographic information, and valid/reliable measures of SE, CS and satisfaction [6, 7, 14, 18]. PHC worker SE was assessed with 4 questions [7], with answers on a five-point Likert scale ranging from 5 = “always” to 1 = “never.“ Higher scores indicated higher SE. A study conducted in Iran found Cronbach’s alpha was 0.82 [6]. A checklist was used to assess PHC worker communication performance with clients in seven areas (2 items for starting the session, 6 items for creating a relationship, 3 items for data collection, 2 items for attending to client’s perception of referral source, 3 items for providing information, 2 items for mutual agreement and 4 items for ending the session). Performance of the skill received a score of 2 (yes) whereas not performing the skill was scored 1 (no). Scores on this construct ranged from 22 to 44. Cronbach’s alpha was 0.78 [7] for this measure. The client satisfaction questionnaire [18, 19] consisted of 42 items in 6 domains (8 items for access to services, 6 items for continuity of care, 8 items for humaneness of staff, 5 items for comprehensiveness of care, 5 items for provision of health education, 10 items for effectiveness of service). Responses were evaluated using a 5-point Likert scale from “strongly agree” (= 2) to “strongly disagree” (= -2). Higher and more positive scores indicate more satisfaction. In Iran, face- and content-validity, and reliability were confirmed [14]. Reliability was assessed for SE and CS questionnaires, in 20 health workers; and for service satisfaction questionnaire in 30 clients were similar to the target population in terms of demographic characteristics. Cronbach’s alphas were 0.81, 0.79 and 0.73 for SE, CS, and satisfaction questionnaires, respectively, when considering each questionnaire as a whole. This was calculated using standard statistical package for social sciences (SPSS 19).

Data were collected prior to training. PHC workers reported on SE and demographics, whereas trained observers completed the CS checklist while observing interactions between PHC workers and clients. Clients completed the satisfaction questionnaire via self-report; persons with no or low literacy completed the questionnaire via interview. Staff members assisting with observations/interviews were blind to condition, and clients were blind to condition. All data were collected 3 months following training, except for demographics.

### Intervention and control groups

The training program was designed and held for the intervention group in four 90-minute training sessions. Training methods included: Lecture and question-and-answer sessions to increase awareness and consolidate learning; film screening; role-playing to enhance SE and improve CS; discussion group to improve SE and CS; instruction booklets; and texting key points of effective communication as reminders. The control group received routine training. Typical training is 2 years consisting of course work, and in-service training. Topics cover general, oral and elderly health; problem solving; collaboration; social factors impacting health; human rights; and cultural beliefs.

### Statistical analysis

Data were analyzed via SPSS 19 using chi-square tests for categorical variables, independent sample t-tests and paired t-tests. An independent sample t-test was used to compare the mean scores of CS questionnaires between intervention and control groups. Also, a paired t-test was used to compare the mean scores of CS questionnaires before and after training sessions. A Mann-Whitney was used to compare the mean scores of SE, and satisfaction questionnaires between intervention and control groups. Also, a Wilcoxon was used to compare the mean scores of SE, and satisfaction questionnaires before and after training sessions. Data normality was confirmed using the Kolmogorov-Smirnov test, histograms, and normality of residuals.

### Ethics

 The Research Ethics Committee of the Saveh University of Medical Sciences approved the study protocol (Number: IR.SAVEHUMS. REC1396.16). Also, all participants in this research completed a written informed consent.

## Results

From 364 service recipients, 358 (180 in the intervention group and 178 in the control group) who completed the post-test underwent the final analysis. The mean age of service recipients was 40.5 ± 14.9 years in the intervention group, and 37.7 ± 12.3 years in the control group (*p* > 0.05). Intervention and control groups were similar on demographic variables (e.g., gender, insurance, education and occupation) and no significant differences were found between groups. Among PHC workers, intervention and control groups were similar on demographic variables (e.g., gender, work experience and literacy level) and there were no significant differences between groups. See Tables 1 and 2.

**Table 1:** Comparison of categorical variables in clients seen by two groups of primary healthcare workers (Behvarz) assigned tobk Intervention and Control groups

Variables	Intervention (***n***=180)		Control (***n***=178)		***P***-value
	Number	Percentage (%)	Number	Percentage (%)	
**Sex**					
Male	79	43.9	82	46	0.67
Female	101	56.1	96	54	
**Education**					
Illiterate	15	8.4	11	6.2	0.54
Elementary	99	55	92	51.7	
High school and diploma	46	25.6	57	32	
Academic	20	11	18	10.1	
**Job**					
Student	8	4.4	10	5.6	0.39
Farmer / Shepherd	43	23.9	54	30.3	
Staff	7	3.9	5	2.9	
Housewife	90	50	88	49.4	
other	32	17.8	21	11.8	
**Insurance**					
Yes	169	93.9	170	95.5	0.49
No	11	6.1	8	4.5	

Note: Chi-square used

**Table 2:** Comparison of categorical variables in primary healthcare workers (Behvarz) assigned to Intervention and Control

Variables	Intervention (***n***=60)		Control (***n***=44)		***P***-value
	Number	Percentage (%)	Number	Percentage (%)	
**Sex**					
Male	25	41.6	16	36.4	0.58
Female	35	58.4	28	63.6	
**Education**					
Elementary	8	13.3	6	13.6	0.12
Middle school	11	18.3	5	11.4	
High school and diploma	33	55	32	72.7	
Academic	8	13.3	1	2.3	
**work experience**					
<10	15	25	9	20.4	0.66
10-19	19	31.7	12	27.3	
≥20	26	43.3	23	52.3	

Note: Chi-square used

According to Table 3, for PHC workers, there was no significant difference between the intervention and control groups before training on SE and all CS constructs except for attending to client perception of referral source (*p* < 0.05). Following training, paired t-tests indicated that the mean scores of SE and all communication skill constructs significantly increased in the intervention group (*p* < 0.001), while mean scores in the control group increased on starting a session, decreased on data collection and evidenced no other significant differences.

**Table 3:** Comparison of communication skills and self-efficacy in primary healthcare workers (Behvarz) assigned to Intervention and Control at baseline and 3-months follow-up

Variable	Group Time	Intervention group Mean ± SD (*N* = 60)	Control group Mean ± SD (*N* = 44)	*P*-value*
Starting the session	Baseline	2.52 ± 0.62	2.38 ± 0.51	0.06
	3-months follow-up	3.79 ± 0.46	2.87 ± 0.72	0.001
	*P*-value**	0.001	0.001	
creating a relationship	Baseline	8.95 ± 1.57	9.0 ± 1.54	0.68
	3-months follow-up	11.91 ± 1.78	9.75 ± 2.03	0.001
	*P*-value**	0.001	0.06	
data collection	Baseline	5.0 ± 0.78	4.84 ± 0.63	0.18
	3-months follow-up	5.51 ± 0.56	4.55 ± 0.49	0.001
	*P*-value**	0.001	0.04	
attending to client perception of referral source	Baseline	3.25 ± 0.89	2.84 ± 0.75	0.01
	3-months follow-up	3.78 ± 0.48	2.77 ± 0.62	0.001
	*P*-value**	0.001	0.39	
providing information	Baseline	4.67 ± 0.75	4.59 ± 0.87	0.56
	3-months follow-up	5.49 ± 0.64	4.69 ± 0.61	0.001
	*P*-value**	0.001	0.55	
mutual agreement	Baseline	2.87 ± 0.56	2.84 ± 0.73	0.95
	3-months follow-up	3.21 ± 0.58	2.66 ± 0.64	0.001
	*P*-value**	0.001	0.27	
ending the session	Baseline	5.64 ± 1.0	5.37 ± 0.85	0.06
	3-months follow-up	6.81 ± 0.92	5.66 ± 1.10	0.001
	*P*-value**	0.001	0.17	*P*-value***
Self-efficacy	Baseline	31.52 ± 2.91	31.32 ± 2.64	0.67
	3-months follow-up	34.25 ± 4.0	31.26 ± 4.52	0.001
	*P*-value****	0.001	0.79	

* Independent T-test

** Paired T-test

*** Mann-Whitney

**** Wilcoxon

For service recipients (Table 4), there were no significant differences between intervention and control groups before training on components of satisfaction (access to services, continuity of care, humaneness of staff, comprehensiveness of care, provision of health education, effectiveness of service), but mean scores of satisfaction variables generally significantly increased in the intervention group after training (*p* < 0.001). No significant differences were observed in the control group from pre- to post-training.

**Table 4:** Comparison of client satisfaction in two groups of primary healthcare workers (Behvarz) assigned to Intervention and Control at baseline and 3-months follow-up

Variable	Group Time	Intervention group Mean ± SD (*N* = 180)	Control group Mean ± SD (*N* = 178)	*P*-value*
Access to services	Baseline	1.75 ± 0.60	1.73 ± 0.47	0.59
	3-months follow-up	2.89 ± 1.0	1.80 ± 0.56	0.001
	*P*-value**	0.001	0.32	
continuity of care	Baseline	1.19 ± 0.97	1.33 ± 0.87	0.28
	3-months follow-up	2.72 ± 1.14	1.40 ± 1.06	0.001
	*P*-value**	0.001	0.34	
humaneness of staff	Baseline	1.22 ± 0.84	1.18 ± 0.79	0.63
	3-months follow-up	2.88 ± 1.17	1.23 ± 0.82	0.001
	*P*-value**	0.001	0.28	
comprehensiveness of care	Baseline	-1.09 ± 0.67	-1.04 ± 0.60	0.14
	3-months follow-up	-1.70 ± 1.22	-0.61 ± 1.14	0.001
	*P*-value**	0.001	0.11	
provision of health education	Baseline	1.05 ± 0.89	1.01 ± 0.95	0.69
	3-months follow-up	2.25 ± 1.12	1.07 ± 0.94	0.001
	*P*-value**	0.001	0.10	
effectiveness of services	Baseline	1.0 ± 0.89	1.10 ± 0.90	0.85
	3-months follow-up	2.75 ± 1.0	1.18 ± 1.0	0.001
	*P*-value**	0.001	0.47	

* Mann-Whitney

** Wilcoxon

Clients were generally dissatisfied with comprehensiveness of care (Table 4). There were no differences between intervention and control groups prior to training. In the intervention group, clients became more dissatisfied with comprehensiveness of care following training; however, no difference was found from pre- to post-training for clients in the control group. Following training, clients in the intervention group were significantly more dissatisfied with comprehensiveness of care than those in the control group. Medians and Interquartile Range of SE and all satisfaction constructs reported in Table 5.

**Table 5:** Comparison of client satisfaction and self-efficacy median in two groups of primary healthcare workers (Behvarz) assigned to Intervention and Control at baseline and 3-months follow-up

Variable	Group Time	Intervention group Median( IR) (*N* = 180)	Control group Median( IR) (*N* = 178)
Access to services	Baseline	2(2)	2(1)
	3-months follow-up	3(3)	2(1)
continuity of care	Baseline	1(2)	1(2)
	3-months follow-up	2.5(1.75)	1(2)
humaneness of staff	Baseline	1(1)	1(1)
	3-months follow-up	3(2)	1.2(1.5)
comprehensiveness of care	Baseline	-1(1)	-1(2)
	3-months follow-up	-2(2)	-1(2)
provision of health education	Baseline	1(0)	1(0)
	3-months follow-up	2(3)	1(0.5)
effectiveness of services	Baseline	1(2)	1(2)
	3-months follow-up	3(4)	1(1.75)
Self-efficacy	Baseline	30(10)	30(10)
	3-months follow-up	32(10)	30(10)

## Discussion

The present study aimed to evaluate the impact of PHC worker training on: (1) PHC worker CS and SE, and (2) client satisfaction with services. Satisfaction among clients of trained PHC workers generally increased from pre- to post-training; and following training, satisfaction generally improved among clients of trained PHC workers as compared to clients of non-trained PHC workers. Similarly, SE and CS increased among trained PHC workers from pre- to post-training; and following training, SE and CS improved among trained PHC workers as compared to non-trained PHC workers. Results indicate training has the potential to improve PHC efficacy and communication skills, and to generally improve client satisfaction with services.

Whereas training seemed to enhance client satisfaction with services across most subscales (e.g., services access, care continuity, staff humaneness, provision of health education, services effectiveness), following training, clients rated comprehensiveness of services with more dissatisfaction. More research should be done to understand elements of training (targeting clinician communication/ confidence) that may adversely impact ratings on comprehensiveness of care in particular. It may be that attending to new communication processes during client interactions distracts clinicians from attending to the range of client health needs. Such an effect might abate over time as clinicians grow accustomed to deployment of communication skills.

Consistent with our overall findings, previous studies emphasized the importance of CS in promoting patient health and satisfaction [20, 21]. For instance, in a study by Boissy et al., [22]communication skill training increased patient satisfaction and improved empathy and SE among physicians. In another study by Bank et al., [23] teaching CS to physicians led to a marked improvement in patient satisfaction. Moore et al., [24] also found that CS training was effective in promoting physical and mental health, satisfaction, and quality of life in patients. Barth and Lannen [25] conducted a meta-analysis and concluded that CS training for healthcare workers is essential for changing their communication behavior and attitudes.

Unlike our findings, a systematic review by Barth and Lannen [25] showed that communication skills of professionals can be improved; yet, patients do not necessarily give higher satisfaction score. In another study by Shilling et al., [26] teaching CS to physicians did not have a significant effect on patient satisfaction. Differences between findings of this study and other studies [26, 27] may be due to differences in client samples. For example, in Schilling et al. [27] service recipients were cancer patients whereas in the present study clients were primary health care recipients.

In our study, SE was specifically targeted for enhancement during CS training (e.g., through discussion, use of role-plays). Previous studies emphasized SE as an important factor for successful performance [27–29], and the current study is consistent with those findings. After the CS training, health worker SE increased significantly in the present study. In a study by Nørgaard et al. [30], and consistent with our findings, health worker SE for communicating with patients increased significantly after CS training. In a review study by Berkhof et al., [31] teaching CS to clinical staff improved patient satisfaction, self-esteem, and SE in doctors. There may be a positive and significant association between CS and SE in clinical staff; therefore, designing successful training to enhance patient-professional communication may be facilitated by attention to staff SE [7]. A study by Cegala DJ and Lenzmeier indicated that applying effective strategies to enhance self-efficacy in medical staffs could lead to satisfaction in both medical staffs and their clients [32]. Considering the results of previous studies [28, 29], and our findings, this could be inferred that designing self-efficacy-based interventions to establish effective communication between medical staff and their clients might be of critical importance. This issue should be considered in developing in-service training for health professionals by authorities.

### Limitation and future directions

The randomized design of this research is a strength, but additional limitations should be considered. Results should be replicated in physicians, nurses, midwives and other health professionals. Statistically, no control was used for factors that may influence outcomes, including PHC worker or client demographics. Nesting within site or PHC worker was also not performed. Alphas were not corrected for family-wise error, but given the consistency and magnitude of the expected effects, results are likely replicable. In addition, formal mediational analyses were not performed to ascertain if the impact of training on client satisfaction is mediated by PHC worker communication or efficacy or both. Future studies may wish to conduct follow-up beyond 3 months to determine whether results enhance or diminish over time, and whether booster training may be appropriate. Future work with extended follow-up might determine client outcomes such as symptom reduction, or program outcomes such as staff turn-over and client drop-out.

## Conclusions

Communication skills training improved the self-efficacy of PHC workers to effectively communicate with clients, improved PHC worker communication skills with clients, and improved clients’ services satisfaction. Findings are encouraging, and such training may be deployed in other practice settings, since it was delivered in only 4 group sessions of 90 min each.

## Data Availability

The datasets used and/or analyzed during this study are available from the corresponding author upon reasonable request.

## Abbreviations

PHC: Primary Healthcare
CS: Communication Skills
SE: Self-Efficacy
